# Astrocytic Regulation of Glutamate Transmission in Schizophrenia

**DOI:** 10.3389/fpsyt.2018.00544

**Published:** 2018-11-06

**Authors:** Yu-Ying Mei, Dong Chuan Wu, Ning Zhou

**Affiliations:** ^1^Translational Medicine Research Center, China Medical University Hospital, Taichung, Taiwan; ^2^Graduate Institute of Biomedical Sciences, China Medical University, Taichung, Taiwan

**Keywords:** schizophrenia, glutamate, NMDA receptors, excitatory amino acid transporters, glutamate-glutamine cycle, D-serine

## Abstract

According to the glutamate hypothesis of schizophrenia, the abnormality of glutamate transmission induced by hypofunction of NMDA receptors (NMDARs) is causally associated with the positive and negative symptoms of schizophrenia. However, the underlying mechanisms responsible for the changes in glutamate transmission in schizophrenia are not fully understood. Astrocytes, the major regulatory glia in the brain, modulate not only glutamate metabolism but also glutamate transmission. Here we review the recent progress in understanding the role of astrocytes in schizophrenia. We focus on the astrocytic mechanisms of (i) glutamate synthesis via the glutamate-glutamine cycle, (ii) glutamate clearance by excitatory amino acid transporters (EAATs), (iii) D-serine release to activate NMDARs, and (iv) glutamatergic target engagement biomarkers. Abnormality in these processes is highly correlated with schizophrenia phenotypes. These findings will shed light upon further investigation of pathogenesis as well as improvement of biomarkers and therapies for schizophrenia.

## Introduction

Dysregulation of glutamatergic neurotransmission is critically implicated in the pathophysiology of psychotic disorders. In the central nervous system (CNS), glutamate is the principal excitatory neurotransmitter and mediates the fast excitatory transmission by activation of ionotropic glutamate receptors, including AMPA, kainate, and NMDA receptors (NMDARs). Astrocytes affect glutamatergic transmission in several important ways, including glutamate biosynthesis, glutamate-glutamine cycle, glutamate uptake, and releasing glutamate and D-serine as gliotransmitters. This review focuses on recent advances in astrocyte-mediated dysregulation of glutamate transmission in schizophrenia.

## The glutamate hypothesis of schizophrenia

The “glutamate hypothesis of schizophrenia” proposes that schizophrenia symptoms and cognitive impairment are due to hypofunction of NMDARs and excessive glutamate release, especially in brain areas including prefrontal cortex and hippocampus (1). This theory was initiated from the observation that NMDAR antagonists, like phencyclidine and ketamine, could evoke negative symptoms and cognitive dysfunction resembling schizophrenic phenotypes in healthy subjects (2). In schizophrenic subjects, subanesthetic doses of ketamine could exacerbate psychotic and cognitive symptoms (3, 4). Interestingly, in encephalitis patients, the first-episode psychosis is associated with the presence of anti-NMDAR antibodies that could cause a reduction of surface expression of NMDARs (5, 6). In animal studies, suppression of NMDAR function by pharmacological or genetic approaches leads to schizophrenia-like behaviors (7, 8). Schizophrenia is also associated with dysregulation of some genes and/or proteins involved in glutamate transmission (9). Genome-wide association studies have reported that the NMDAR subunits encoding genes, GRIN2A and GRIN2B, are schizophrenia-related genes (9, 10). Two *de novo* mutations in GRIN2A were found in sporadic schizophrenia patients (11). The single nucleotide polymorphisms of genes related to D-serine synthesis and metabolism, such as genes encoding serine racemase (SR), D-Amino Acid Oxidase (DAAO), and the DAAO activator G72, are also associated with schizophrenia (12).

**Figure 1:**
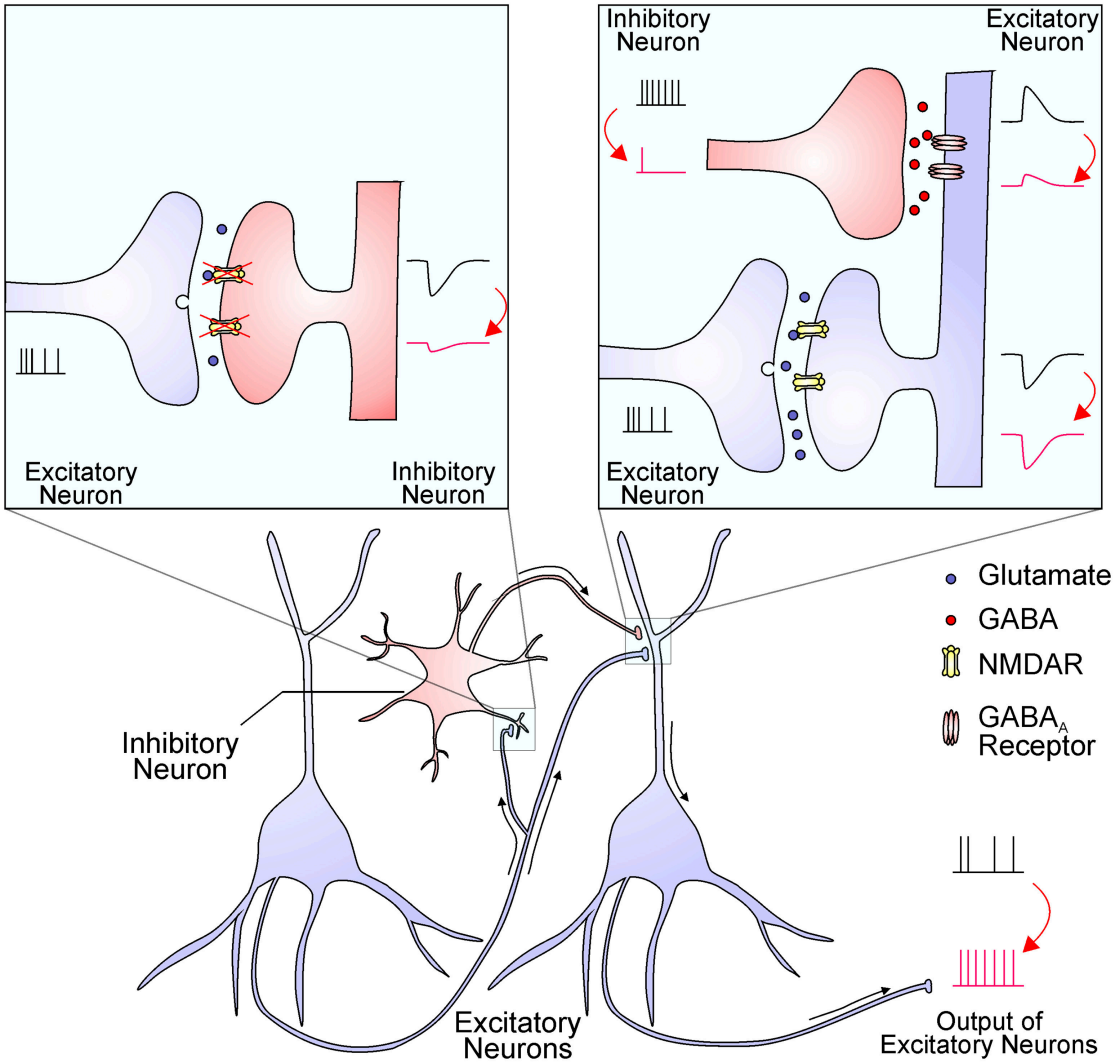
The NMDAR hypofunction hypothesis proposes that NMDARs in inhibitory interneurons are preferentially diminished in schizophrenia. The reduced NMDAR function in interneurons (the left inset) results in decreased excitatory postsynaptic currents, which in turn decrease interneuron output (the right inset). The reduced GABA release from interneuron terminals leads to smaller inhibitory postsynaptic responses and thereby a disinhibition of postsynaptic excitatory neurons (the right inset). This finally leads to an enhanced output of excitatory neurons, resulting in excessive glutamate release and increased activation of non-NMDA glutamate receptors. This process might be enhanced by NMDAR antagonists and ameliorated by mGluR2/3 agonists.

The hypofunction of NMDAR causes excessive glutamate release, hyper-glutamatergic functions, and hypermetabolism (13). However, it remains largely unknown how NMDAR hypofunction leads to elevated glutamate levels and over-activation of non-NMDA glutamate receptors. One possibility is that interneurons are more sensitive to NMDAR blockade than principal glutamatergic neurons, and interneuron inhibition leads to an overall effect of disinhibition and hyperexcitation of glutamatergic output (Figure 1) (1, 14, 15). The increased glutamate levels have been documented by brain imaging studies and the source of excessive glutamate is mainly from presynaptic release (16). This notion was strengthened by the effect of drugs that specifically reduce presynaptic glutamate release, like the mGluR2/3 agonists, which significantly reverse ketamine-evoked glutamate elevation and cerebral blood volume increase in the hippocampus (13). High extracellular glutamate levels lead to hypermetabolism, structural disorganization, and eventually hippocampal volume reduction, and these dysfunctions are correlated with disease progression from prodromal symptoms to psychosis (17). Nevertheless, many mechanisms that cause an overall increase in glutamate tone could contribute synergistically to schizophrenia pathogenesis.

## Synaptic and extrasynaptic NMDARs

**Figure 2:**
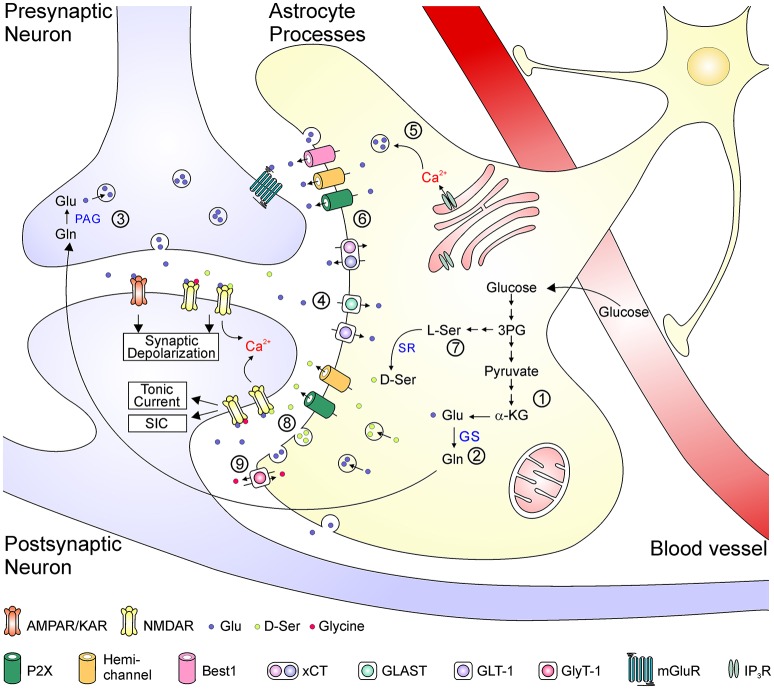
Schematic depiction of the astrocyte-related mechanisms in the metabolism, transport, uptake of glutamate and the regulation of glutamate transmission. Glutamate is synthesized *de novo* from α-KG via the TCA cycle (1). In the glutamate-glutamine cycle (2–4), glutamate is metabolized to glutamine by GS in the astrocyte and then transported to the neuron (2). Neuronal PAG catalyzes the conversion from glutamine into glutamate, which is released as the transmitter from presynaptic terminal (3). The synaptically released glutamate is rapidly uptake by EAATs back into the astrocytes (4). Glutamate can be released as a gliotransmitters by either Ca^2+^-dependent vesicular release (5) or efflux from transporters or large-conductance ion channels (6). D-serine is converted from L-serine, which is synthesized *de novo* from 3-phosphoglycerate in astrocytes (7). D-serine can be released via either vesicular or non-vesicular mechanisms and activate neuronal NMDA receptors (8). Glycine is transported across astrocytic membrane via glycine receptors (9). 3PG, 3-phosphoglycerate; α-KG, α-ketoglutarate; Glu, glutamate; Gln, glutamine; GS, glutamine synthetase; L-Ser, L-serine; D-Ser, D-serine; PAG, phosphate-activated glutaminase; SR, serine racemase; ASCT, alanine-serine-cysteine transporter; xCT, cystine-glutamate antiporter; GLAST, glutamate aspartate transporter; GLT-1, glutamate transporter 1; GlyT-1, glycine transporter 1; mGluR, metabotropic glutamate receptor; IP3R, inositol trisphosphate receptor.

NMDARs are distributed at both synaptic and extrasynaptic sites with different subunit compositions. Their activation requires the binding of both glutamate and D-serine/glycine as a co-agonist. Synaptic NMDARs are activated by presynaptic glutamate release at low frequency, while intensive synaptic activation could lead to glutamate spillover and activation of extrasynaptic NMDARs (18). Synaptic NMDARs are important for synaptic plasticity and learning and memory, which is largely controlled by D-serine concentration within the synaptic cleft depending on astrocyte coverage and function (19, 20). Moreover, extrasynaptic NMDARs are in close proximity to synapse-enveloping astrocytes and are a preferential target for astrocyte-released glutamate, D-serine, and glycine (Figure 2). Activation of extrasynaptic NMDARs could generate two forms of responses. First, ambient glutamate could produce a tonic NMDAR current, whose amplitude is directly modulated by glial EAATs (21, 22). The tonic NMDAR currents contribute to neuronal excitability and their existence has been identified in multiple neuron types in the prefrontal cortex, hippocampus, and cerebellum (23–25). Second, activation of extrasynaptic NMDARs could produce “slow inward currents” (SICs), which are infrequent phasic events characterized by slow activation and decay kinetics, large amplitude, and insensitivity to sodium channel blockers. It is believed that SICs are due to astrocyte-originated glutamate and the co-occurrence of SICs in adjacent neurons could lead to synchronized neuronal activity (26). It is also possible that SICs are generated by the phasic release of D-serine/glycine, which potentiates extrasynaptic NMDARs that are already tonically activated by low concentrations of ambient glutamate (18). While accumulating evidence has demonstrated a critical role of deficient synaptic NMDARs in schizophrenia (27, 28), alterations in the expression and function of extrasynaptic NMDARs are less understood. It was recently reported that the tonic NMDAR conductance was significantly larger and more sensitive to NMDAR blockers in interneurons compared with pyramidal neurons in the hippocampus (29). However, it remains to be determined whether extrasynaptic NMDAR deficiency occurs under schizophrenic conditions.

## Astrocytes and glutamate transmission

Regulation of glutamate transmission could occur at several steps, including synthesis, transportation, release, and clearance of glutamate, as well as activation of glutamate receptors by endogenous agonists and co-agonists. Figure 2 summarizes some key mechanisms underlying the impact of astrocytes in glutamate transmission in the CNS. Astrocytes are the primary locus for the biosynthesis of glutamate from glucose. Through the tricarboxylic acid cycle, the glycolysis product pyruvate is converted into α-ketoglutarate, which is then catalyzed into glutamate by aspartate aminotransferase (30). Glutamate is catalyzed by glutamine synthetase (GS), which is exclusively expressed in astrocytes in the brain, to form glutamine that is further transferred to neurons. In glutamatergic neurons, glutamate is converted from glutamine by phosphate-activated glutaminase (30). After packaged into synaptic vesicles, glutamate is released as the neurotransmitter to active postsynaptic AMPA/kainate receptors to mediate fast excitatory synaptic transmission, and to activate NMDARs to depolarize the postsynaptic membrane potential and to allow Ca^2+^ influx. The clearance of excessive glutamate from the synaptic cleft occurs rapidly, largely due to the function of four subtypes of EAATs (EAAT1-4), among which the EAAT1 (also termed glutamate-aspartate transporter, GLAST) and EAAT2 (also termed glutamate transporter 1, GLT-1) are mainly expressed in astrocytes (31). Once glutamate is taken up into astrocytes, it is converted into glutamine and re-enters the glutamate-glutamine cycle. Besides their key roles in the biosynthesis of glutamate and maintenance of the glutamate-glutamine cycle, astrocytes can directly release glutamate as a gliotransmitters by either Ca^2+^-dependent vesicular exocytosis or by non-vesicular release (32, 33). Astrocytes are also a major source of glycine and D-serine to potentiate NMDAR responses. Glycine is stored in astrocytes with high concentrations (3–6 mM) and can be released via the reversed operation of glycine transporters and other Ca^2+^-independent pathways (34–36). The synthesis of D-serine is initiated from the glycolytic intermediate metabolite 3-phosphoglycerate, which is synthesized into L-serine in several steps. D-serine is converted from L-serine by the action of SR and is released by Ca^2+^-dependent and/or -independent machineries from astrocytes (19, 37) [but see (38)]. Notably, these above processes might interact with one another; for example, the increased glutamate synthesis leads to elevated glutamate release (39), and activation of glutamate transports could evoke rapid glutamine release as a feedback mechanism (40).

## EAATs

The efficiency of glutamate clearance directly affects extracellular resting glutamate levels and kinetics of glutamate-mediated synaptic responses. Among the total four subtypes of brain-expressed EAATs, EAAT1, and EAAT2 are mainly expressed in astrocytes, and EAAT2 is the dominant transporter in adults and is responsible for about 90% of total glutamate uptake (41, 42). The EAATs are mainly responsible for clearing synaptic glutamate and shaping postsynaptic responses. Deficits in EAAT functions cause the persistently increased glutamate levels, which represent one of the most remarkable changes in the schizophrenic brain. Accumulating evidence has reported abnormal mRNA and/or protein expression levels of EAATs in the prefrontal cortex, hippocampus, and anterior cingulate cortex in the postmortem tissue from schizophrenia patients. While many studies have revealed an overall reduction of EAAT2 levels in schizophrenia, some other studies reported unchanged or increased EAAT2 levels in certain brain regions [for review see (43)]. In a recent study using the laser-capture microdissection technique to separately harvest glutamatergic relay neurons and astrocytes from the mediodorsal nucleus of thalamus, it was reported that changes of EAAT levels could be cell-specific: mRNA expression of EAAT1 was decreased in astrocytes, whereas EAAT2 was increased in excitatory relay neurons in schizophrenia (44). The upregulation of EAATs in neurons is possibly due to a compensatory response to the reduced EAAT levels in astrocytes. Notably, the mRNA levels of EAAT2b were remarkably increased in anterior cingulate cortex pyramidal neurons and thalamus relay neurons in schizophrenia (44, 45). EAAT2b is a splicing variant of EAAT2. It contains the PDZ domain-binding motif and possibly interacts with neuronal scaffolding proteins like PSD-95 (45–47). The EAAT2b proteins are localized predominantly at the cell surface and their distribution can be regulated by intracellular Ca^2+^ and CaMKII (48), suggesting that EAAT2b might exhibit neuronal specific functions.

The abnormal EAAT levels are highly associated with schizophrenia phenotypes. Mice lacking EAAT1 displayed schizophrenia-like behavioral changes: they showed higher locomotor activity in the novel environment but not in the home cage, and had increased sensitivity to the locomotor hyperactivity induced by NMDA antagonists (49). The locomotor hyperactivity of EAAT1 knockout mice was reversed by either the antipsychotic haloperidol or the mGlu2/3 agonist LY379268 (49). These mice also exhibited abnormalities in behavioral tests that measure the negative and cognitive symptoms of schizophrenia, including poor nesting behaviors, lesser preference for novel social stimulus albeit normal overall social interaction, a significant reduction in acoustic startle amplitude, and impaired learning in an instrumental visual discrimination task, suggesting that EAAT1 dysfunction could generate certain behavioral changes resembling schizophrenia phenotypes (49, 50). Knockout of EAAT2 caused lethal spontaneous seizures, high susceptibility to brain injury, and short life-span in the homozygous mice (51). The heterozygous EAAT2 knockout mice had less severe abnormalities, and they exhibited increased locomotion activity in a new environment and greater freezing responses in the fear-conditioning test compared to the wildtype (52). The EAAT1/2 double knockout mice showed multiple brain defects resembling developmental defects found in schizophrenia (53). In a recent study, the inducible EAAT2 knockout mice were generated in a temporal controlled, cell-specific manner. The study demonstrated that knockout during adolescence caused 60–80% reduction of EAAT2 selectively in astrocytes, and the animals showed no lethal seizures or neuronal loss. Interestingly, these animals exhibited pathological repetitive behaviors, like excessive self-grooming and tic-like head shakes, whereas they had no obvious anxiety or social abnormality (54).

It remains unknown how EAAT abnormality leads to schizophrenia-relevant behaviors. In hippocampus, pharmacological inhibition of EAAT functions increased the peak amplitude of NMDAR-mediated EPSCs and prolonged their decay kinetics, but had no changes in AMPA receptor-mediated EPSCs, possibly due to the fast desensitization of AMPA receptor currents (55). It has been generally considered that EAATs could limit glutamate spillover to neighboring synapses and enhance synapse independence, which is related to synaptic plasticity as well as learning and memory (31). Reduced expression of glial EAATs will not only impair these important physiological functions but also increase the susceptibility of the brain to injury and cell death (51). Neuronal EAATs might have much lower efficiency than the astrocytic transports: glutamate is quickly converted into glutamine by GS, resulting in low free glutamate concentrations in astrocytes; whereas neurons are liable to accumulate intracellular glutamate concentration and thereby lead to poor operation of neuronal EAATs. Moreover, the action of EAATs will produce a non-stoichiometrically coupled anion current (56, 57), which contributes to the regulation of neuronal membrane potentials and transmitter release (58). In the zebrafish photoreceptor synapse, the EAAT2b isoform mediates a large-conductance Cl^−^ current, potentially capable of affecting resting membrane potentials (59). Nevertheless, these mechanisms remain to be determined in schizophrenia and may vary depending on the brain region and disease progression.

## The glutamate-glutamine cycle

Schizophrenia is associated with the abnormal glutamate-glutamine cycle. Altered levels of brain metabolites including glutamate, GABA, and glutamine have been well-documented in human studies and these alterations can be detected in different brain regions and the cerebrospinal fluid (CSF) (60). Researchers using proton magnetic resonance spectroscopy (^1^H-MRS) have reported abnormal glutamine and/or glutamate levels (some studies report glutamate+glutamine levels in combination depending on the ^1^H-MRS approach and field strength), especially in the brain regions involving the medial temporal lobe (including hippocampus), basal ganglia, and thalamus of schizophrenia patients (61). An elevation of the glutamate+glutamine level was also observed in hippocampus of healthy humans receiving ketamine administration (62). The CSF glutamine/glutamate ratio is higher in the first-episode patients compared with normal controls (63). GS, the key enzyme involved in the glutamate-glutamine cycle, exhibits lower protein levels in spite of increased expression of mRNA in schizophrenia; but the results were less consistent among studies [for review see (43)]. Elevated glutamine levels are also directly related to psychotic symptoms and neuropsychological tests (64, 65). Although changed glutamine levels are supposedly related to functional alterations of astrocytes, it is notable that the ^1^H-MRS and CSF data cannot provide a precise measure of intracellular vs. extracellular substrates and further evidence is needed to establish the causality.

Disturbance of the glutamate-glutamine cycle and down-regulation of GS have also been reported in human epilepsy (66, 67). Reduced GS activity will build up intracellular glutamate in astrocytes, leading to reduced uptake capacities of EAATs. Moreover, glutamine produced by astrocytic GS is also one of the major sources for maintaining synaptic vesicle content of GABA in inhibitory interneurons (68). Pharmacological inhibition of GS reduced inhibitory postsynaptic currents in hippocampal pyramidal neurons and was reversed by exogenous application of glutamine (68). Similar dysfunction of the inhibitory synaptic response was also found in neurons near reactive astrocytes, which showed profound decreases in GS expression levels (69). These effects increased network hyperexcitability and produced spontaneous recurrent seizures (70). The loss of interneurons and deficient cortical GABA synthesis are also one of the most robust pathologies of schizophrenia (71–73). The reduced parvalbumin interneurons are associated with impaired oscillatory activity in hippocampus and imbalance of cortical excitation-inhibition (72, 74). Impaired parvalbumin interneuron activity by selective deletion of ErbB4 or dopamine D2 receptors produced schizophrenia-like behaviors in mice (75, 76). Dysfunction of neuron-astrocyte interaction has been suggested in schizophrenia models (77). However, it remains unclear whether there is any causative relationship between the dysfunction of the glutamate-glutamine cycle and interneuron deficiency.

## D-serine

D-serine plays an important role in the dysregulation of NMDAR functions in schizophrenia (78). This was supported by several lines of evidence, including the reduced serum and CSF D-serine levels (79, 80), a genetic association of SR and DAAO (the enzyme responsible for the degradation of D-serine) polymorphisms in schizophrenia patients (12, 81), the reversal effects of D-serine on schizophrenia-like behaviors in animals (82), and the beneficial effects of clinical D-serine-targeting therapies in patients (83, 84).

Recent studies revealed that the abnormality in astrocyte-released D-serine plays a critical role in the DISC1-associated pathological processes. The DISC1 gene was identified from patients with familial mental disorders and has been considered a critical susceptibility gene for schizophrenia (85, 86). The density of DISC1-expressing astrocytes is largely reduced in the dentate gyrus of hippocampus in schizophrenia patients compared with healthy controls (87). Selective expression of mutant DISC1 in astrocytes decreased protein levels of SR by increasing its ubiquitination (88). Astrocytes expressing mutant DISC1 caused less elaborated dendritic arborization and decreased the density of excitatory synapses in co-cultured normal neurons (89). The downregulation of SR resulted in a significant decline in D-serine levels, an enhanced locomotor activity, and impaired responses to pre-pulse inhibition in the mutant mice (88). Mice carrying the astrocytic specific mutation of DISC1 also exhibited increased anxiety, attenuated social interaction and preference for social novelty, and impaired cognitive behaviors (90). These behavioral abnormalities in DISC1 mutant mice were reversed by treatments of D-serine (88, 90), indicating a strong correlation with NMDAR hypofunction.

The α7 subunit-containing nicotinic acetylcholine receptor (α7 nAChR) has been considered a strong genetic contribution to schizophrenia (91). It has been reported that activation of α7 nAChR contributes to dopamine release and induction of long-term potentiation (91). Interestingly, α7 nAChRs are expressed not only in neurons but also in astrocytes (92–94). ACh could induce the intracellular Ca^2+^ elevation in astrocytes by activation of α7 nAChRs and facilitate the synthesis and release of D-serine (94–97). Papouin et al. recently discovered that astrocytic α7 nAChRs activation could drive the vesicular release of D-serine from astrocytes to generate a wakefulness-dependent D-serine oscillation *in z* (98). The wakefulness and activity but not the circadian rhythms of the animal promote D-serine fluctuations over the 24 h period, during which D-serine was accumulated during the wakefulness and declined during sleep. Such an oscillation of the D-serine level is associated with NMDAR activity and the learning and memory behavior of the animal, consistent with the involvement of NMDARs in the cognitive deficits in schizophrenia. Astrocytes appear to play a central role since D-serine accumulation during the wakefulness is impaired by disruption of astrocytic vesicular exocytosis machinery or by selective deletion of α7 nAChRs from astrocytes rather than neurons. More importantly, EVP-6124, an α7 nAChR modulator tested in schizophrenia clinical trials, is able to promote D-serine release and enhance NMDAR activity (98).

## Potential biomarkers

Several types of translational biomarkers have been developed to assess prognosis and monitor disease progression and treatment in schizophrenia. First, functional magnetic imaging (fMRI) blood-oxygenation-level dependent response (BOLD) has been applied to test brain physiology following acute NMDAR antagonist administration. NMDAR antagonists evoke robust changes in relative cerebral blood volume and local metabolism, which is largely due to the energy consumption for increased glutamate release and uptake (13, 99). The second approach applies ^1^H-MRS to measure glutamine and/or glutamate levels in the brain (16, 100). As described above, abnormal glutamine and/or glutamate indices were found in brain regions of individuals with schizophrenia compared with healthy volunteers (61). The third method utilizes task-based fMRI to evaluate BOLD responses in hippocampus and dorsolateral prefrontal cortex (101). The most adapted task is item-specific encoding task, which is designed to assess contributions of specific encoding and retrieval processes to episodic memory (102). Schizophrenia patients with more severe negative symptoms exhibited poor BOLD responses during the encoding and retrieval of episodic memory (101, 103). And fourth, positron emission tomography (PET) technique has been used to evaluate glutamatergic, GABAergic, and monoamine neurotransmission in various brain functions and have established the association between these responses and cognitive functions in schizophrenia (104, 105). Finally, the severity of schizophrenia symptoms is associated with abnormal serum levels of several glutamate transmission related factors, including brain-derived neurotrophic factor (BDNF), vascular endothelial growth factor (VEGF), D-serine, G72, and multiple inflammatory factors (106–108). A recent clinical study by Javitt et al. compared the first three types of glutamatergic target-engagement biomarkers in healthy volunteers receiving ketamine or placebo infusions (109). The data revealed a significant increase in the NMDAR antagonist-evoked BOLD responses. The ^1^H-MRS results showed a smaller but significant change in glutamate+glutamine levels. However, the data from task-based fMRI failed to yield a significant difference between the ketamine and placebo group (109). These observations indicate the fMRI BOLD as a particularly relevant biomarker for NMDAR antagonist-induced responses. The potential application of one or combined biomarkers for more refined prognostic classification of schizophrenia remains to be further assessed.

## Future treatments

Based on the glutamate hypothesis, many drug targets have been proposed and some are under investigation. For example, drugs targeting the co-agonist binding site of NMDARs are of the primary interest [for review see (110)]. Direct enhancement of NMDAR functions with glycine and D-serine has been used in multiple clinical trials, among which one recent study showed improved negative symptoms in individuals at clinical high risk of schizophrenia (111). GlyT1 inhibitors, like sarcosine or Bitopertin, which are supposed to increase synaptic glycine levels, have been found effective in several trials (112). Other treatments to increase D-serine/glycine levels, like the DAAO inhibitor sodium benzoate, have demonstrated beneficial effects as an add-on drug (83, 84). The mGluR2/3 agonists have been shown to inhibit excessive glutamate release in animal studies, and the tested drug LY2140023 has produced significant improvement in patients (113). Moreover, the α7 nAChR has attracted much attention; several drugs targeting this receptor are under clinical investigation (110). Besides these, more specific regulation of certain proteins in the glutamate transmission, such as selective NMDAR subtypes or EAAT isoforms, might represent potential targets for future drug development.

## Conclusions

The glutamate hypothesis has substantially advanced our understanding of the pathogenesis of schizophrenia. Although glutamate transmission is mediated by ionotropic glutamate receptors and involves other pre- and post-synaptic components at excitatory synapses, astrocytes regulate glutamate metabolism and shape glutamate transmission in several important aspects. This review has focused on some recent studies that explicitly report the important role of glutamate uptake, glutamate-glutamine cycle, and astrocyte-derived D-serine in the etiology of schizophrenia. Neuroimaging and neurochemical biomarkers based on glutamate transmission and metabolism have also demonstrated translational utility. Further understanding of neuron-astrocyte interaction during these processes will be critical to the development of diagnostic and therapeutic avenues for schizophrenia and psychotic disorders.

## Author contributions

All authors listed have made a substantial, direct and intellectual contribution to the work, and approved it for publication.

### Conflict of interest statement

The authors declare that the research was conducted in the absence of any commercial or financial relationships that could be construed as a potential conflict of interest.
